# A Secure Artificial Intelligence-Enabled Critical Sars Crisis Management Using Random Sigmoidal Artificial Neural Networks

**DOI:** 10.3389/fpubh.2022.901294

**Published:** 2022-05-04

**Authors:** Shiwei Jiang, Hongwei Hou

**Affiliations:** School of Politics and Public Administration, Zhenghzhou University, Zhengzhou, China

**Keywords:** severe acute respiratory syndrome coronavirus 2, artificial intelligence, block-matching filter, histogram equalization, compact entropy rate superpixel, histogram of gradient, Principal Component Analysis, Random Sigmoidal Artificial Neural Networks

## Abstract

Since December 2019, the pandemic COVID-19 has been connected to the severe acute respiratory syndrome coronavirus 2 (SARS-CoV-2). Early identification and diagnosis are essential goals for health practitioners because early symptoms correlate with those of other common illnesses including the common cold and flu. RT–PCR is frequently used to identify SARS-CoV-2 viral infection. Although this procedure can take up to 2 days to complete and sequential monitoring may be essential to figure out the potential of false-negative findings, RT–PCR test kits are apparently in low availability, highlighting the urgent need for more efficient methods of diagnosing COVID-19 patients. Artificial intelligence (AI)-based healthcare models are more effective at diagnosing and controlling large groups of people. Hence, this paper proposes a novel AI-enabled SARS detection framework. Here, the input CT images are collected and preprocessed using a block-matching filter and histogram equalization (HE). Segmentation is performed using Compact Entropy Rate Superpixel (CERS) technique. Features of segmented output are extracted using Histogram of Gradient (HOG). Feature selection is done using Principal Component Analysis (PCA). The suggested Random Sigmoidal Artificial Neural Networks (RS-ANN) based classification approach effectively diagnoses the existence of the disease. The performance of the suggested Artificial intelligence model is analyzed and related to existing approaches. The suggested AI system may help identify COVID-19 patients more quickly than conventional approaches.

## Introduction

As of January 5, 2021, the COVID-19 outbreak had resulted in eighty-five million positive cases and over 1.85 million deaths in over a hundred and ninety nations throughout the world, putting it on par with the 1918 flu pandemic, according to several experts. However, it is more pertinent to compare the covid pandemic with the SARS because of the proximity in time, the closeness of the outbreak's origins, the virus's structure, and different administrations' responses. The SARS pandemic began in the Chinese province of Guangdong in November 2002, although the epidemic did not materialize until the spring of 2003. A total of twenty-three persons were infected at Hotel Metropol by a “super-spreader,” a doctor who flew to Hong Kong from Guangdong and stayed at the hotel. People throughout the world were infected by the virus and were labeled a pandemic by the WHO. In the period between November 1, 2002, and July 1, 2003, 8,445 persons in twenty-six nations were affected with SARS, with approximately 80 percent of the fatalities occurring in mainland China and Hong Kong.

The COVID-19 pandemic, which began in Wuhan, China, toward the end of November, shares certain characteristics with the SARS epidemic but also differs. Since 2003, this type of virus has not been found, and COVID-19 shares eighty percent of its genome with SARS, indicating that both viruses are quite common. It is to blame for a pandemic that has put people's health, livelihoods, and way of life at risk. It's hardly hyperbole to say that medical attention is urgently needed. Healthcare systems in various nations have been severely strained by the pandemic (1). There were 44 cases of human-to-human transmission identified on January 3, 2020, by Chinese national authorities. “Severe acute respiratory syndrome coronavirus 2” (hereinafter SARS-CoV-2) has been given the name by the “International Committee on Taxonomy of Viruses (ICTV).” This virus produces unique coronavirus disease 2019. Even though most SARS-CoV-2 patients exhibit mild symptoms like fever, coughing, or shortness of breath, a large percentage of people go on to develop severe pneumonia in both lungs, which can be fatal. SARS-CoV-2 had been linked to 5.8 million positive cases and over 360,000 deaths as of the end of May 2020. Pandemic status was granted by the WHO in March 2020, although experts believe the organization should have forewarned the public sooner (2).

**Figure 1:**
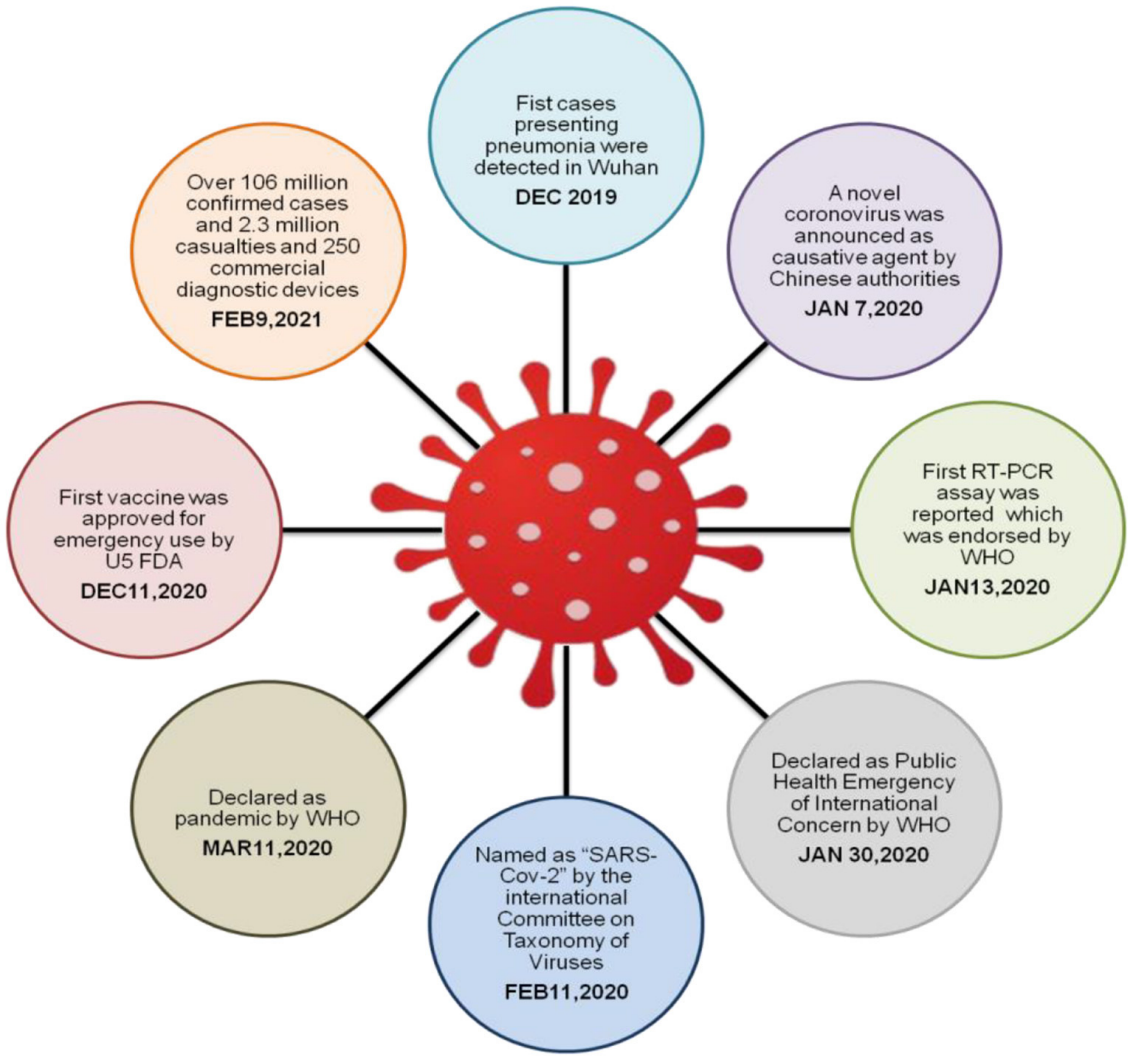
Timeline of COVID-19.

People's concerns about the sickness and how it's being dealt with are growing. The timeline of COVID-19 is shown in Figure 1. The economic effects of the coronavirus have been felt all around the world, and daily life has been interrupted in several countries as a result. There are severe consequences if the outbreak is not contained and managed as quickly as possible, including a shortage of medical personnel and medical equipment (3). In the absence of specialized COVID-19 treatment medications or vaccinations, it is critical to promptly segregate the infected person from the uninfected population. When a patient is diagnosed with a viral infection, they receive immediate treatment and are isolated to stop the further spread of the disease. Early in the propagation of a virus, RT-PCR testing is regarded as the gold standard. Some problems may arise when the most afflicted countries enter community transmission. Test time and accuracy must be balanced as soon as possible as the virus enters the phase of widespread transmission in communities. Waiting a few days before screening tests would be hazardous to the human body, given a large number of new instances being reported every day. Because of this, a viable alternative technique is urgently required (4). In large populations, healthcare models based on artificial intelligence (AI) are more effective in diagnosing and controlling diseases.

As a result, this research provides a revolutionary SARS detection methodology that is powered by artificial intelligence. The subsequent portion of the investigation justifies the literature study conducted in Section Related Works. Section Proposed Methodology provides a visual representation and detailed description of the suggested method. Section Results and Discussion illustrates the results obtained. Finally, in Section Conclusion, some concluding thoughts have been provided.

## Related Works

In (5) the author found invariant and hypervariable areas in the spike protein by monitoring 527,988 SARS-CoV-2 genomes across time. Mutations from the disease lineages were combined and determined to have the greatest lineage-defining mutations in the N-terminal region. It has been observed that structural features at local mutation sites are considerably changed. The Delta mutant showed extensive intra-protein interactions, in contrast to the sparse connections seen in wild-type proteins. Consequently, the author believes that major structural changes in spike proteins have occurred, which may affect the pathophysiology and need the repositioning of vaccine candidates.

In (6) the author mentions that biomedical researchers must have accurate information regarding SARS-CoV-2 and COVID-19. Viruses have a wealth of biological information, however, integrating that knowledge is challenging and time-consuming because much of it is in siloed databases or text. KG-COVID-19 is a versatile framework that integrates diverse biological information to develop “Knowledge Graphs (KGs).” For future pandemics, the KG framework may be used to swiftly combine siloed biomedical data from many sources for varied research purposes.

In (7) the author depicts that a combination of “Deep learning (DL)” and “Multi-Level Feature Extraction (MLFE)” is used to achieve automated coronavirus detection in CT scans and chest X-rays. To extract information from images, this technique uses an MLFE methodology that includes GIST, “Scale Invariant Feature Transform (SIFT),” and a CNN. It's the goal of MLFE to lower the training difficulty of a CNN, which greatly aids in COVID-19 detection. The retrieved COVID-19 characteristics are detected using “Long Short-Term Memory (LSTM)” in conjunction with a CNN network.

In (8) the author illustrates that MOF materials are being used in the battle against the current worldwide pandemic scenario connected to the corona illness to combat SARS-CoV-2. The author outlines the uses and possibilities of MOF materials in this struggle. The major focus is on certain MOF compounds, their crystal structures, textural qualities, and the possibility of modification and application in coronavirus SARS-CoV-2 diagnostics and eradication.

In (9) the author mentions that SARS-CoV-2 has been identified as a possible pharmacological target using an integrated drug repositioning framework that utilizes ML and analysis techniques to explore large-scale graphs, related works, and transcriptome data. For the treatment of COVID-19, a “poly-ADP-ribose polymerase 1 (PARP1)” now in Phase I trials, CVL218, may be an option.

In (10) the author explains that during the duration of the pandemic, SARS-CoV-2 strains undergo constant evolution and gain mutations in their genomes. Because of these genetic traits, an infection may be more severe. These mutations have been shown to have a significant impact on the COVID-19 illness in hospitalized patients. By using a “Directed Acyclic Graph (DAG),” assumptions about the link between (confusing) variables can be explicitly expressed in a causal model. It is possible to examine different study designs and the potential for selection bias by presenting a variety of DAGs.

In (11) the author investigates COVID-19 identification from CT lung images will be investigated using transfer learning. Several transfer learning architectures are tested, as well as the impacts of HE and “Contrast Limited Adaptive Histogram Equalization (CLAHE),” according to the findings.

In (12) the author offers an end-to-end system for the identification of coronavirus from CT scans that includes deep extraction and selection. They use three CNNs for feature extraction. The meta-heuristic optimization technique “Harmony Search (HS)” is paired with the local search method “Adaptive-Hill Climbing (AHC)” for improved performance. There are 2,482 CT images in the CT-Scan data, as well as an improved version of the earlier 2,926 CT-Scan data.

In (13) the author explains that an elevated cytokine response is the cause of cytokine release syndrome in COVID-19. Therapeutic plasma exchange (TPE) may be able to enhance clinical outcomes by removing these cytokines before the improvement of end-organ damage. Patients with severe COVID-19 may benefit from the use of TPE as a preliminary guide document. TPE's participant in the treatment of corona patients was examined to determine the best course of action.

In (14) the author depicts that the COVID-19 epidemic has spawned the most severe worldwide health catastrophe in recent times. Many severely sick COVID-19 patients demonstrate a dysregulated host response, because of this death rates are quite high in this population. Patients with COVID-19 may benefit from extracorporeal cytokine elimination, as it has in other cases of hyper inflammation. Early intervention with the CytoSorb blood purification device can stabilize the hemodynamic and enhance key organ functioning. This is the most extensively researched cytokine elimination platform. The goal of their study is to offer an overview of the pathophysiology of hyper inflammation in COVID-19 and to highlight the existing research on the effects of hem adsorption in these individuals.

In (15) the author examines the course of coronavirus in a pregnant woman, the clinical problems of patient treatment in the critical care setting, and the recommended multidisciplinary methods using a technique to improve maternal and newborn results.

In (16) the author mentions that the COVID-19 crisis management difficulties have a significant impact on the importance and potential of resilience in the COVID-19, according to this study. It has become increasingly difficult to maintain global peace as the second wave of the COVID-19 contagious sickness spreads rapidly over the world. To stop, maintain, and maintain the local spread of the corona, public health officials took active action and suggested that health professionals take several preventative safety measures. Behavioral and interventional alterations that may be indicative of changes in the infection kinetics of the COVID-19 are examined in this study. Global healthcare issues and crisis management are discussed as well as two systems interventional approaches that can assist curb the spread of the coronavirus by using practical crisis management preventative measures to lessen the load on a health monitoring system.

In (17) the author illustrates that the massive burden on the healthcare system has been caused by a continuing epidemic of COVID-19 that originated in China's Hubei province. These patients' care is continuously being refined. COVID-19 individuals who are severely sick are the focus of their observational cohort research. Patients' histories, treatments, and short-term outcomes were collected daily by doctors working at the bedside. The author goes through how this hospital approaches to therapy, including recommended severity scale.

In (18) the author covers crucial issues about “patient screening,” “environmental controls,” “personal protective equipment,” “resuscitation procedures,” and “critical care unit operations” planning as they are ready for additional cases or local outbreaks of 2019-nCoV. No matter how many 2019-nCoV cases the author can only hope that the lessons learned from previous infectious disease crises will enhance the level of preparation.

In (19) the author mentions that comorbidities linked with SARS-CoV-2 sickness and mortality were a primary focus of the research. “PRISMA (Preferred Reporting Items for Systematic Reviews and Meta-analyses)” criteria guided the design of the research. Only 28 papers met the inclusion criteria from a total of 1726 research. It is estimated that among the 28 trials, 5,686 people had SARS-CoV-2 positive infected ranging from moderate to high. There were 17 fatalities among the 108 patients who required a ventilator for severe/critical illness. For 48 of the 108 ventilated children, a medical history was provided. The pre-existing cardiac illness was found in 11/48 individuals, making the overall comorbidity rate for the group of 48 patients. It was stated in 12 of the 17 individuals who died that they had a prior medical history. Comorbidities accounted for 75% of the cases.

In (20) the author depicts that worldwide individuals and healthcare systems might be at risk because of the SARS-CoV-2 pandemic spread. Older folks, those with many medical conditions, and those who are malnourished in general had the lowest results and the highest fatality rates, according to research. These findings are based on the most recent research and are geared at patients who are not in an intensive care unit (ICU) or have any medical conditions that are known to increase the risk of malnutrition.

In (21) the author discussed SARS-CoV-2 antiviral effectiveness *in vitro*. Patients with this disease may benefit from the use of these medications based on their *in vitro* database, side effects, and possible toxicities.

In (22), many of the disorders have multiple odontogenic keratocysts. A 12-year-old female youngster had several odontogenic keratocysts. The studies found no other anomalies indicative of a condition.

In (23), personalized medicine employs fine-grained data to identify specific deviations from normal. These developing data-driven health care methods were conceptually and ethically investigated using “Digital Twins” within engineering. Physical artifacts were coupled using digital techniques which continuously represent their state. Moral differences can be observed based on data structures and interpretations imposed on them. Digital Twins' ethical & sociological ramifications are examined. The Healthcare system has become increasingly data-driven. This technique could be a social equalizer by providing efficient equalizing enhancing strategies.

In (24), allergic rhinitis would be a long-standing worldwide epidemic. Taiwanese doctors commonly treat it with either traditional Chinese or Chinese–Western drugs. Outpatient traditional Chinese medicine therapy of respiratory illnesses was dominated by allergic rhinitis. They compare traditional Chinese medicine with western medical therapies treating allergic rhinitis throughout Taiwan.

In (25), the usage of high-dose-rate (HDR) brachytherapy avoids radioactivity, allows for outpatient therapy, and reduces diagnosis timeframes. A single-stepping source could also enhance dosage dispersion by adjusting latency at every dwell location. The shorter processing intervals need not permit any error checking, and inaccuracies could injure individuals, hence HDR brachytherapy therapies should be performed properly.

In (26), this study presented a treatment as well as the technology of domestic sewage to improve the rural surroundings.

In (27), soil samples from chosen vegetable farms throughout Zamfara State, Nigeria have been tested for physicochemical & organochlorine pesticides. Testing procedure and data were analyzed using QuEChERS with GC-MS.

### Problem Statement

To properly contain the spread of SARS-CoV-2 within a community, WHO recommends early testing for the virus that causes illness. In contrast to serology tests, both RT-PCR and direct viral antigen testing can diagnose the disease quickly in the course of the disease. The pandemic, on the other hand, has placed significant pressure on the supply chain, which has made it difficult to efficiently execute a population-scale case detection activity based on RT-PCR. Although well-funded health care systems have been able to respond effectively, the lack of proper RT-PCR testing capacity has hampered their efforts. The situation is considerably more serious in low and middle-income nations, with screening rates that are orders of magnitude lower than those in high-income ones.

## Proposed Methodology

To promptly identify COVID-19 positive patients in the early stages of SARS-CoV-2 infection, we set out to develop an AI system that can detect SARS-CoV-2 infection based on initial chest CT scans.

**Figure 2:**
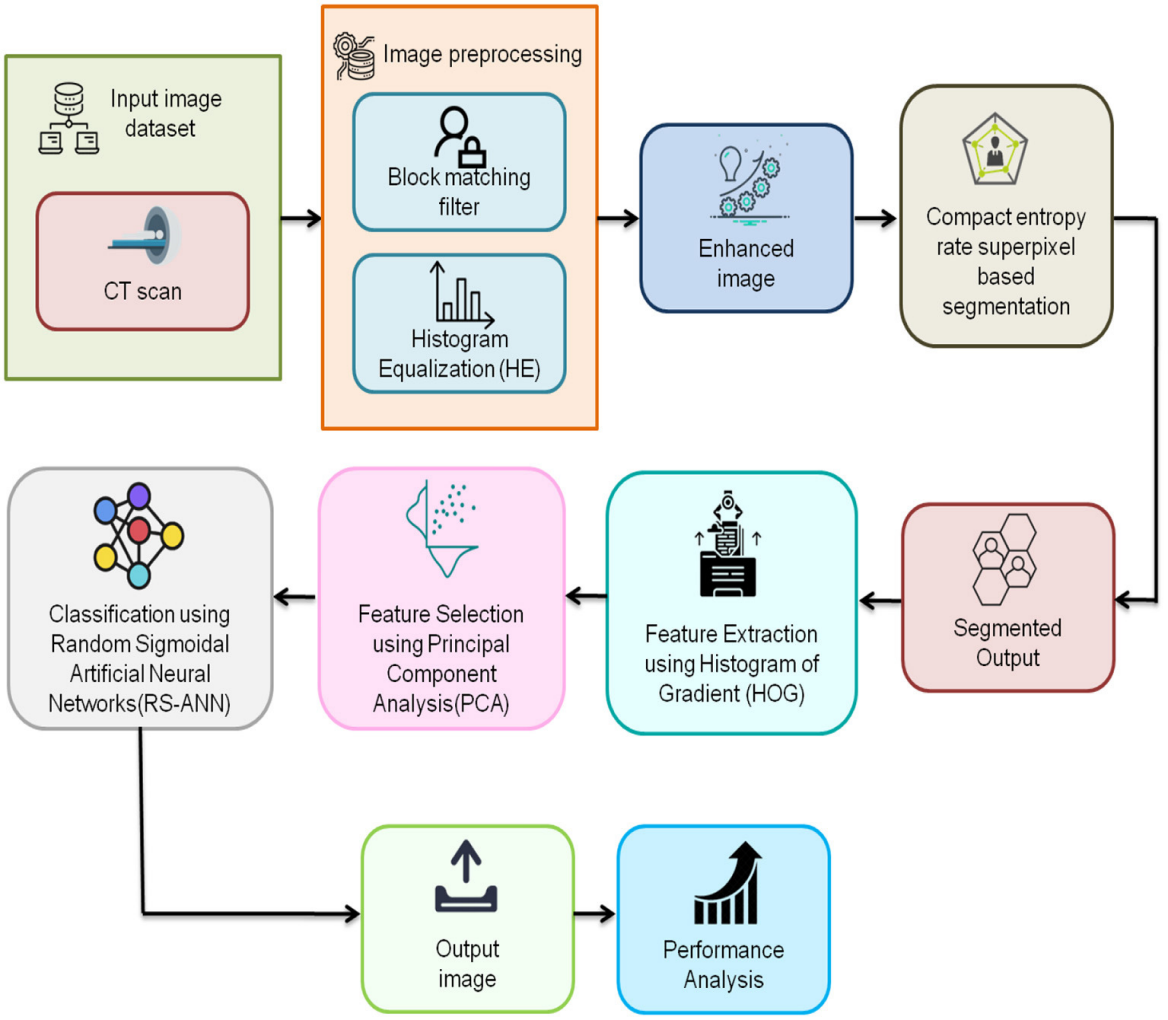
Schematic representation of the suggested methodology.

Figure 2 depicts the flow of the suggested methodology. The input CT images are collected and preprocessed using a block matching filter and histogram equalization. Then the enhanced image is segmented by using the compact entropy rate superpixel technique. Segmented output features are extracted using a histogram of gradient. Feature selection is done using Principal Component Analysis (PCA). The proposed Random Sigmoidal Artificial Neural Networks (RS-ANN) based classification approach effectively diagnoses the existence of the disease.

### Input Image Dataset

Raw hospital data received from GitHub repositories were used in our study (COVID-19-tracker, 2020). CT images were employed as the data source for this study. Hospitals and clinics keep records of patients' information in an anonymized form when they come in for diagnosis and treatment. Six thousand, five hundred and twelve people from seven distinct provinces in China were included in our datasets. Haoming Xu, a Chinese researcher, and native speaker, confirmed the accuracy of the dataset after it was translated into Mandarin Chinese using Google Translator. Beijing University's Big Data High-accuracy Center conducted a study that provided the data utilized in this article. As of the 20th of February 2020, these statistics were gathered through the COVID-19-tracker. The patient's characteristics are shown in Table 1 (27).

**Table 1:** Patient characteristics.

**Information**	**All patient *n* = 6,512 No. (%)**	***P*-value**
**Demographic information**		
Age, median	42 (32–54)	<0.002
**Gender**		
Male	3,365 (51.71)	-
Isolation treatment	1,412 (21.6)	<0.001
Travel history	4,238 (65.2)	<0.002
**Symptoms**		
Cough	1,976 (30.2)	<0.002
Fever	2,674 (41)	<0.001
Muscle soreness	25 (0.5)	0.9181
Runny nose	548 (8.42)	<0.002
Lung infection	854 (12.99)	<0.002
Diarrhea	38 (0.58)	0.3489
Pneumonia	488 (7.49)	<0.001
**Have symptoms**	2,970 (46.5)	-

### Image Preprocessing

When preprocessing CT image, the aim is to improve the quality of the data by suppressing undesired distortions and enhancing some aspects that are crucial for subsequent processing.

#### Block Match Filter

Gray distribution is used in the block matching process to compare a single-pixel gray value with the neighboring one. However, when the image features or edges are prominent, comparing the grayscale distribution results in an inaccurate categorization. The most comparable blocks are found by combining pixel-based similarity and structure-based similarity. For this purpose, we assume that the image's fixed-size blocks exhibit mutual correlation. The nearest block to the reference block is selected when correlation matching is tested at the pixel level, as seen in this example. The correlation coefficients are obtained and hard thresholding is conducted on blocks that are comparable to the reference block, which is then arranged in 3D. Hard thresholding results in the image are divided into two portions, and correlation coefficients are obtained without any noise. When stacked in an array, there is a direct correlation between the size of the blocks and how well they match. The only way to decode a 3D decoration is to contract the 3D conversion coefficients, which rarely represent the underlying signal. With a higher “peak signal-to-noise ratio (PSNR),” the compressed or rebuilt image will have better quality. Using an equation, the PSNR value is determined (1) (Algorithm 1).

**Algorithm 1:** Random Sigmoidal Artificial Neural Network (RS-ANN).

w1, w2 ← Random number from [−1, 1]
w1- Hidden layer weight, w2 - Output layer weight
No. of. Correct = 0
for i < No. of. TI do
for j < size (train) do
Inp (j) ← train (j)
HO (j) ← f [B; w1, Inp (j)]
Out (j) ← g [Bias; w2, HO (j)]
if Out (j) = TL (j) then
No. of. Correct+ = 1
end if
del_1_ ← [Out (j) − TL (j) * (1 − out (j)^2^]
del_2_ ← (W2 * delta) * [1 − HO (j)^2^]
w1 ← w1 − alpha * [Inp(j) * del_2_]
w2 ← w2 − alpha * [HO(j) * del_1_]
end for
accuracy ← No. of. Correct /n
end for
if w1 > 0 then
w1 ← 1
else
w1 ← 0
end if
if w2 > 0 then
w2 ← 1
else
w2 ← 0
end if

When evaluating the quality of image compression, the “Mean Square Error (MSE)” and the PSNR are employed as metrics. MSE is calculated with the help of Equation (2). Although the MSE depicts the total cumulative squared error between the compressed and original images, PSNR represents a measure of the peak error between both images. Error decreases as MSE decreases.


(1) $$\begin{array}{l} PSNR=10loglog_{10}\left(\frac{MAX_{m}^{2}}{MSE}\right) \end{array}$$

(2) $$\begin{array}{l} MSE=\frac{1}{no}\sum_{j=0}^{n-1}\sum_{k=0}^{0-1}{\left[J\left(j,k\right)-L\left(j,k\right)\right]}^{2} \end{array}$$

#### Histogram Equalization (HE)

Noise removed CT images are further preprocessed using HE. Other elements, such as light and other circumstances, will have an impact on the collection of CT images. As a result, the quality of the CT images taken will be uneven, and the contrast between the images will below. Image contrast will be raised, and image quality will be improved, as a result of the use of image HE. The image data that has been processed is kept as input for the neural network.

To begin, we calculate the probability distribution of each gray level in a grayscale image.

There are a total of T pixels in the image, and there are a total of *Q*(*g*_*j*_) instances of the jth gray level frequency, hence *g*_*j*_ indicates the gray level and *o*_*j*_ indicates the number of occurrences.


(3) $$\begin{array}{l} Q\left(g_{j}\right)=\frac{o_{j}}{T},j\in \left(0,1,2,\ldots ,255\right) \end{array}$$

Cumulative distribution function is calculated by using Equation (4)


(4) $$\begin{array}{l} O_{l}=\sum_{j=0}^{l}Q\left(g_{j}\right) \end{array}$$

After the gray level, remember *h*_*k*_ for the HE, and then


(5) $$\begin{array}{l} h_{k}=INT\left[\left(h_{max}-h_{min}\right)T_{l}+h_{min}+0.5\right] \end{array}$$

Rounding operation is denoted by INT. HE is a mapping of *h*_*k*_ and *g*_*j*_. After the equalization, the greyscale of the image is more uniformly distributed, increasing the contrast.

#### Segmentation

Using the compact entropy rate superpixel (CERS) approach, the increased image features are segmented. A superpixel is an area of the image that is seen to be uniform. To accommodate the image's borders, this approach puts together pixels with similar color and textures. The CT image's superpixel is a good place to look for features since it depicts real-world objects. Using a CT image graph representation, the random walk entropy is combined with an averaging term for the segment size. A high entropy leads to homogenous regions in the graph. The asymptotic measure of residual random process uncertainty after adding edges to form clusters is known as the entropy rate. The objective function is submodular and the best solution is within 1/2 of the bounds shown by this group. Edges that provided the most profit were included in their algorithm first. These stages were continued until the desired number of clusters were formed. To speed up the process, CERS omitted the edges that may create cycles. This procedure was able to limit the number of possible solutions.

#### Feature Extraction

There are two major steps in the HOG algorithm for extracting features. The first step is to extract the oriented gradient's histogram. Each pixel in the input CT image is used to derive the gradient's magnitude and direction information. These are used to create an angle histogram of gradients, which is then used as a feature vector for an image texture. At the pixel level, the vertical and horizontal components of the image K(k, l) are derivatives from one another. They are calculated in the following ways, respectively:


(6) $$\begin{array}{l} Hk\left(k,l\right)=K\left(k+1,l\right)-K\;\left(k-1,l\right) \end{array}$$

where,


(7) $$\begin{array}{l} HL\left(k,l\right)=K\left(k,l+1\right)-K\;\left(k,l-1\right) \end{array}$$

and,


(8) $$\begin{array}{l} H\left(k,l\right)=\sqrt{Hk{\left(k,l\right)}^{2}+Hl{\left(k,l\right)}^{2}} \end{array}$$

and,


(9) $$\begin{array}{l} \alpha_{0}\left(k,l\right)=tan^{-1}\left[\frac{G_{l}\left(k,l\right)}{G_{k}\left(k,l\right)}\right],\alpha_{0}\in \left[\frac{-\pi }{2},\frac{\pi }{2}\right] \end{array}$$

A horizontal and vertical derivative of *Hk*(*k, l*) *and Hl*(*k, l*) at pixel (k, l) are shown. The gradient of the CT image is used to generate the HOG descriptor in the second stage. To begin, the entire image is divided into 8^*^8 chunks. The gradient direction range [- $\frac{\pi }{2}$, $\frac{\pi }{2}\;$] is divided into nine equal intervals (bins). HOG feature findings are normalized by dividing each bin by the sum of the histogram to provide a strong vector to brightness changes.

#### Feature Selection

By using highly discriminative features, it is possible to improve the classifier's classification accuracy. Our feature extraction method was PCA because of its simplicity, effectiveness, and popularity as the oldest multivariate method. Analysis of dependent and inter-correlated factors in the real image is performed using PCA, a multivariate statistical process that transforms these variables into a new collection of variables known as principal components. It is possible to obtain a new set of principle components, each with a certain variance, with the first principal component having the biggest variance among the other principal components. The information quantity that may be maintained is highly dependent on the selection of principal components; as a result, the maximum information can be retained by selecting an adequate number of principal components to lower the dimensionality of the dataset.

Through the use of linear transformation V(N, M), PCA can reduce a 2-D CT image matrix (where N is the total number of pixels after masking and O is the number of instances such that O < N) to a smaller matrix A(O, M), (where M is the number of pixels such that M < N) while retaining much of the information contained in the data.


(10) $$\begin{array}{l} A=V^{U}Y \end{array}$$

Covariance matrices T (M, M) are computed to represent data in the form of


(11) $$\begin{array}{l} T_{A}=\frac{1}{O}A^{U}A \end{array}$$

An eigenvector equation with a Lagrange multiplier λ maximizes covariance. An algorithm called matrix diagonalization is then used to break down these eigenvector equations into a product of three matrices:


(12) $$\begin{array}{l} T=QEQ^{-1} \end{array}$$

Equivalently, where E refers to the diagonal matrix and Q refers to the matrix of eigenvectors. Therefore, the total of eigenvalues is shown in the equation as the full variance of the transformation (13)


(13) $$\begin{array}{l} Total\;variance=\sum_{j=1}^{N}⋋j \end{array}$$

The variance maintained in the subset of these N vectors is used to project the data from the top M eigenvectors.


(14) $$\begin{array}{l} Retained\;variance=\sum_{j=1}^{M}⋋j \end{array}$$

As a result, Equation (15) expresses the quantity of information kept as a percentage of the original.


(15) $$\begin{array}{l} Percentage\;of\;information\;retained=\frac{\sum_{j=1}^{M}⋋j}{\sum_{j=1}^{N}⋋j} \end{array}$$

A covariance matrix is computed, eigenvector and value pairs are determined using matrix diagonalization. These pairings are then sorted by decreasing the eigenvalue order. Top M pairings will save the most information while consuming just a fraction of their original dimensions since eigenvalues are proportional to the variance maintained.

#### Classification Using Random Sigmoidal Artificial Neural Networks (RS-ANN)

Now the selected features from CT images are classified using a Random sigmoidal artificial neural network (RS-ANN). To simulate linear and non-linear outputs through a learning process, it is built on a computational framework of layers. As illustrated by the feedforward RS-ANN model, local and global approximation may be efficiently coupled. A three-layered structure is employed in its construction. Using the CT image's pre-selected characteristics, the input layer can quickly and accurately identify COVID-19 (+) patients. Activation functions and sigmoid data are used to turn these selected attributes into sigmoid data ranging from 0 to 1. The activation function's broad structure is depicted in Equation (16)


(16) $$\begin{array}{l} t\left(y\right)=\frac{1}{1+e^{-y}} \end{array}$$

From zero to one, this is the range of the sigmoid value *t* (y), which is the independent input value. The total of the information received from the input layer's connections is processed by the neurons in the hidden layers. The activation function then processes the summations and passes the results to the following layer. To the output layer, this procedure is carried on. The sigmoid data in the hidden layer are multiplied with the weights that were previously assumed. The anticipated range of weights was 0–1. The following weights are applied to each node:


(17) $$\begin{array}{l} x_{i}=\sum y_{i}\text{w}+\text{b}\left(+1\right) \end{array}$$

Where x i is the total of each node y i's initial weights w plus bias (b). +1 is the bias used. Addition of bias results in the summation of a number that is not zero. To prevent division by zero in subsequent calculations, it is imperative that 0 be avoided (infinity). To produce the final output, the hidden layer's related coefficients are linked and organized in matrices. Use the pseudo-code below to easily identify situations of positive COVID-19 results.

## Results and Discussion

The performance of the suggested approach is measured using a MATLAB simulation tool. Specifications such as a) Accuracy, b) Precision, c) Recall, and d) F1-score are used to validate the suggested approach's behavior. This evaluation will take into account four factors: *t*_*p*_ denotes true positive, *t*_*n*_ denotes true negative, *f*_*p*_ denotes false positive, and *f*_*n*_ denotes false negative.

- *t*_*p*_ represents the COVID-19 (+) cases are correctly identified as positive.
- *t*_*n*_ represents the COVID-19 (-) cases that are correctly identified as negative.
- *f*_*p*_ represents the COVID-19 (-) cases that are mistakenly identified as positive.
- *f*_*n*_ represents the COVID-19 (+) cases being mistakenly identified as negative.

### Accuracy

It is a metric that shows how many instances were successfully identified compared to the total cases. However, in cases when the distribution of classes is unequal, this statistic may not be an accurate representation of a classifier's real performance. The following formula can be used to determine accuracy:


(18) $$\begin{array}{l} Accuracy\;=\frac{t_{p}+t_{n}}{t_{p}+t_{n}+f_{p}+f_{n}} \end{array}$$

**Figure 3:**
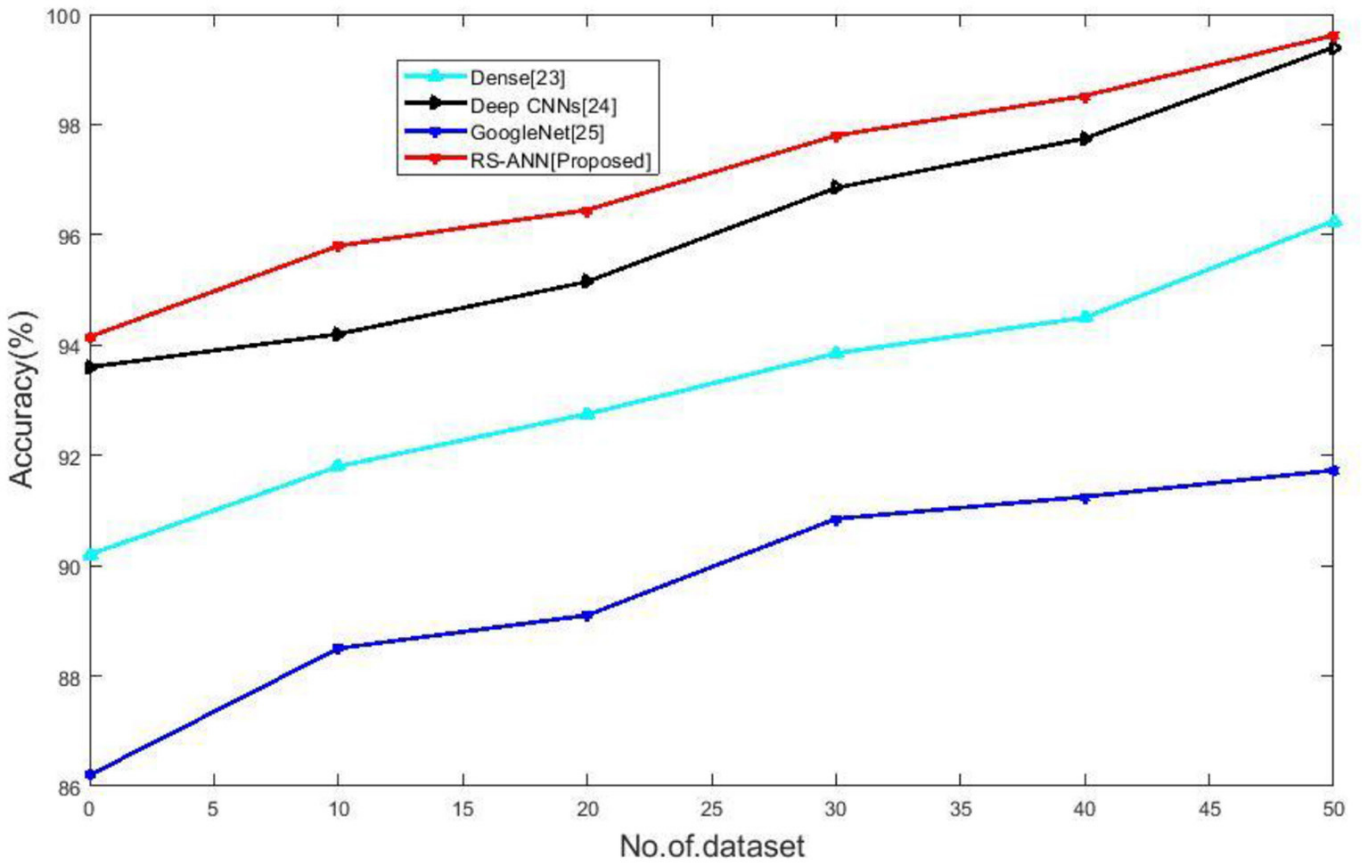
No of dataset vs. accuracy.

Figure 3 compares the accuracy of existing methods such as Dense (28), Deep CNNs (29), Google Net (30) with the proposed RS-ANN. It clearly shows the proposed method achieves 99.62% accuracy which is far better than the traditional methods.

### Precision

In situations when we need to be certain of our predictions, precision is a useful measurement to use. It quantifies the percentage of expected positives that are positive. False positives are more likely to occur when the model's accuracy is low.


(19) $$\begin{array}{l} Precision=\frac{t_{p}}{t_{p}+f_{p}} \end{array}$$

**Figure 4:**
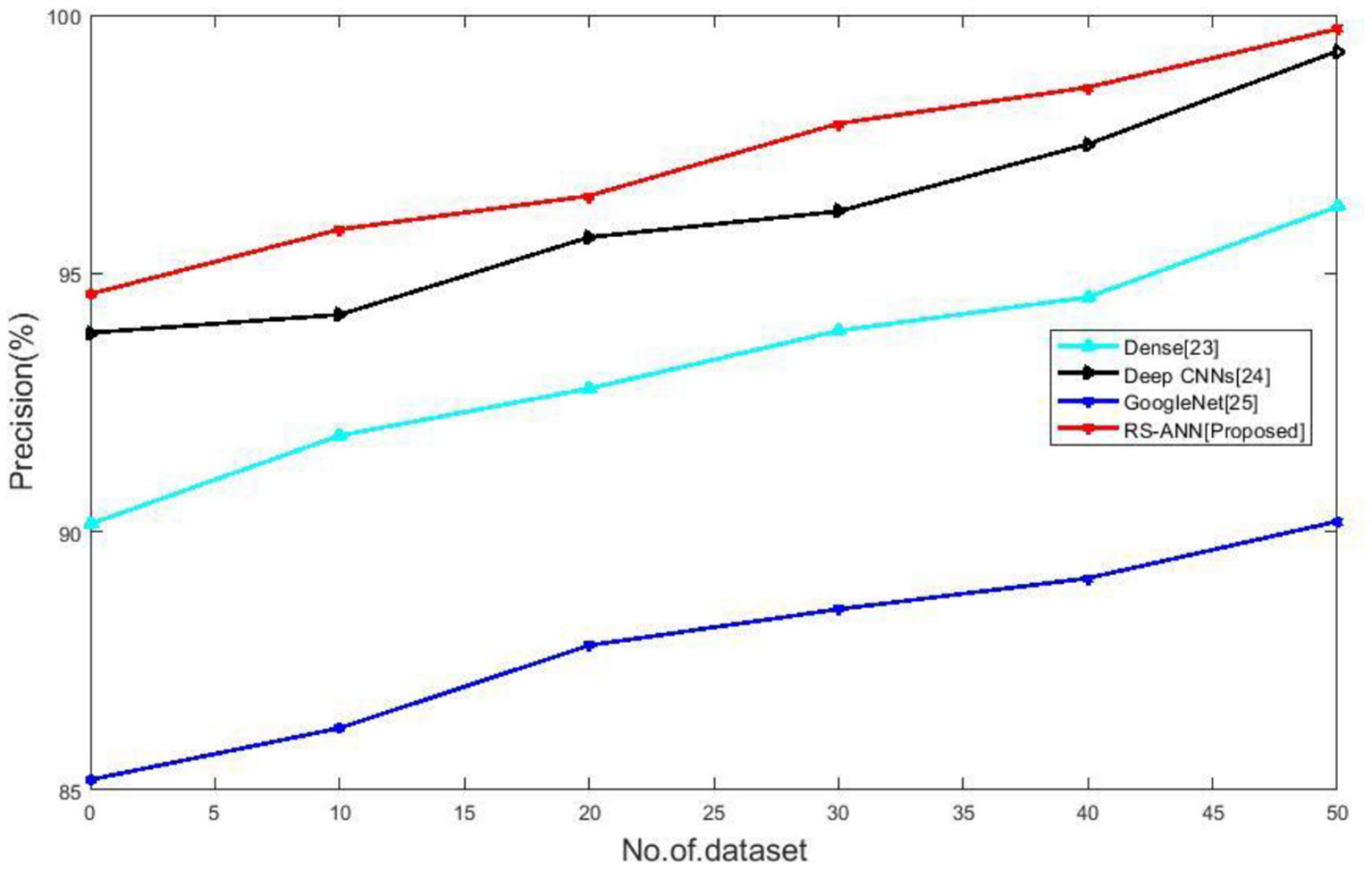
No of dataset vs. precision.

Figure 4 compares the precision of existing methods such as Dense (28), Deep CNNs (29), Google Net (30) with the proposed RS-ANN. It clearly shows the proposed method achieves a precision of 99.73% which is superior to the existing methods.

### Recall

It is defined as the ratio of all successfully categorized positive examples by a model to the total number of true positive instances being tested. It is referred to as sensitivity in some cases. The following formula can be used to calculate recall:


(20) $$\begin{array}{l} Recall\;=\;\frac{t_{p}}{t_{p}+f_{n}} \end{array}$$

**Figure 5:**
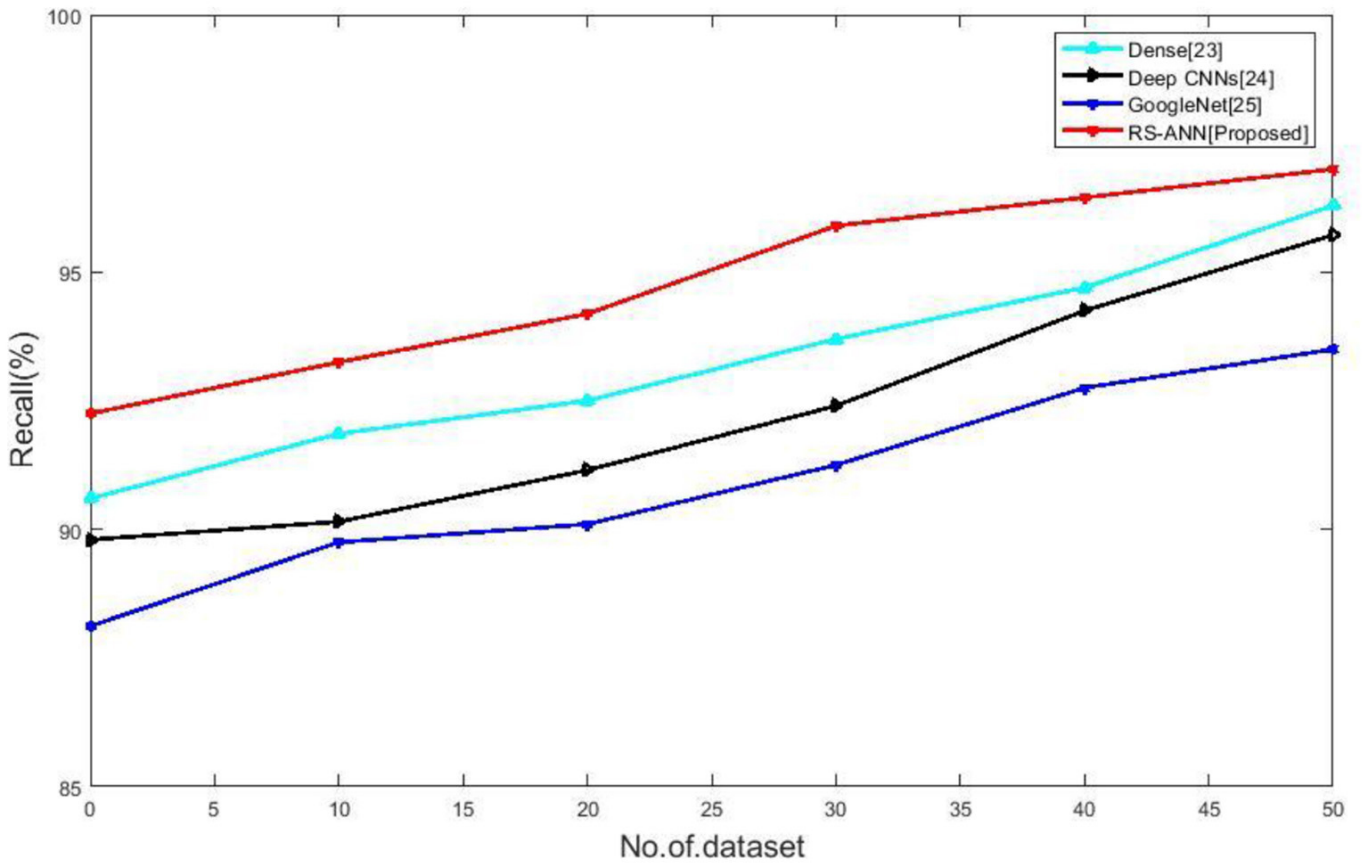
No of dataset vs. recall.

Figure 5 compares the recall of existing methods such as Dense (28), Deep CNNs (29), Google Net (30) with the proposed RS-ANN. It clearly shows the proposed method achieves a recall of 97% is higher than the existing methods.

### F1-Score

As a measure of precision and recall, it's often utilized as a harmonic average. A high F1-score may be achieved when there is a right proportion between precision and recall. One of these variables may be improving at the expense of the other if F1-score is not extremely high.

F1-score can be calculated using the following formula:


(21) $$\begin{array}{l} \text{F}1-\text{score}=\frac{2\times \text{precision}\times \text{recall}}{\text{precision}+\text{reccall}} \end{array}$$

**Figure 6:**
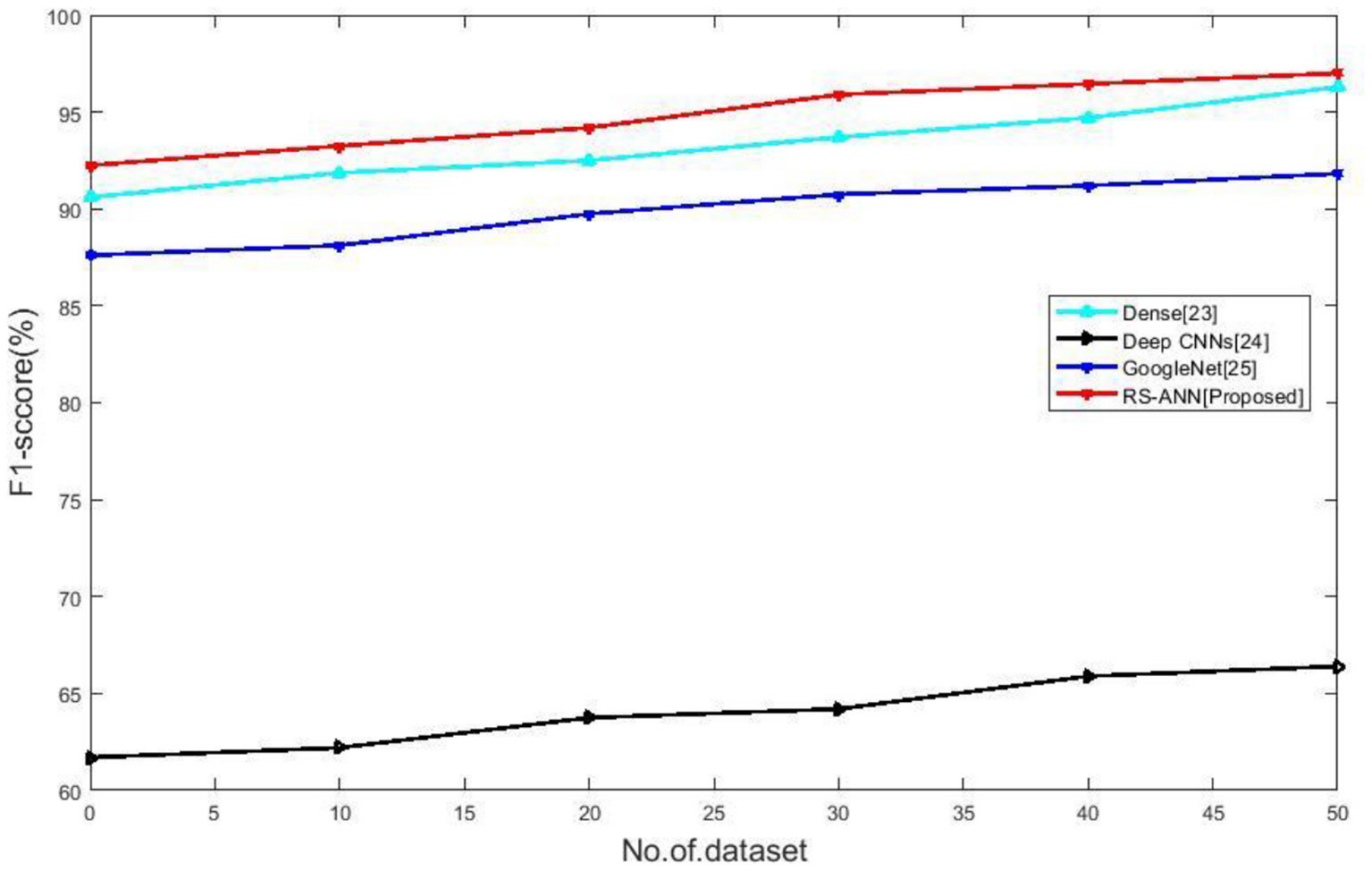
No of dataset vs. F1-score.

Figure 6 compares the F1-Score of existing methods such as Dense (28), Deep CNNs (29), Google Net (30) with the proposed RS-ANN. It clearly shows the proposed method achieves the f1-score of 97 % which is better than the existing methods. A comparison of traditional approaches and new methods is depicted in Table 2.

**Table 2:** Comparison of traditional methods with proposed method.

**Algorithm**	**Accuracy (%)**	**Precision (%)**	**Recall (%)**	**F1-score (%)**
Dense (28)	96.25	96.29	96.29	96.29
Deep CNN (29)	99.4	99.6	94.25	66.4
Google net (30)	91.75	90.20	93.50	91.82
RS-ANN [Proposed]	99.62	99.73	97	97

## Conclusion

The SARS-CoV-2 virus is the most serious public health crisis, affecting people all over the world (31). Reducing the virus's pace of spread is the most effective strategy to halt it. Only by detecting and treating it at an early stage can this be accomplished. Hence, we presented RS-ANN to quickly identify corona virus-positive cases from CT scans. Here the input images are preprocessed using block-matching filter and histogram equalization. CERS technique is used to segment the preprocessed image. A histogram of Gradient and PCA is used to extract and select the segmented features. The suggested method effectively diagnoses the existence of the disease. The performance of the suggested AI system is evaluated and compared with existing approaches in terms of metrics like accuracy, precision, recall, and F1 measure. The whole experimentation was carried out under MATLAB simulation. The suggested model achieves 99.62% accuracy. Compared to other traditional classifiers, our results showed that the RS-ANN system surpassed the accuracy of other classifiers, Dense, Deep CNN, and Google Net, respectively. Future research will focus on how to fully use the semantic information in the images while combining natural language processing to provide a description or image diagnosis report for the COVID-19 CT scan images.

## Data Availability Statement

The original contributions presented in the study are included in the article/supplementary material, further inquiries can be directed to the corresponding author/s.

## Author Contributions

SJ and HH: study conceptualization, design, data collection, data analysis, interpretation, and paper writing. Both authors listed have made a substantial, direct, and intellectual contribution to the work and approved it for publication.

## Conflict of Interest

The authors declare that the research was conducted in the absence of any commercial or financial relationships that could be construed as a potential conflict of interest.

## Publisher's Note

All claims expressed in this article are solely those of the authors and do not necessarily represent those of their affiliated organizations, or those of the publisher, the editors and the reviewers. Any product that may be evaluated in this article, or claim that may be made by its manufacturer, is not guaranteed or endorsed by the publisher.
